# Exercise Perceptions and Experiences in Adults With Crohn’s Disease Following a Combined Impact and Resistance Training Program: A Qualitative Study

**DOI:** 10.1093/crocol/otad019

**Published:** 2023-03-24

**Authors:** Katherine Jones, Jenni Naisby, Katherine Baker, Garry A Tew

**Affiliations:** Warwick Clinical Trials Unit, Warwick Medical School, University of Warwick, Coventry, UK; Department of Sport, Exercise and Rehabilitation, University of Northumbria at Newcastle, Newcastle Upon Tyne, UK; Department of Sport, Exercise and Rehabilitation, University of Northumbria at Newcastle, Newcastle Upon Tyne, UK; School of Science, Technology and Health, York St John University, York,UK

**Keywords:** Crohn’s disease, qualitative, exercise perceptions, resistance training

## Abstract

**Background:**

Exercise is increasingly being recognized to counteract specific complications of Crohn’s disease (CD). The aim of this study was to explore exercise experiences and perceptions after engaging in a combined impact and resistance training program, involving both intervention and control group viewpoints.

**Methods:**

Semistructured telephone interviews, involving a convenience sample of participants with CD (*n* = 41; aged 49.1 ± 12 years) were undertaken up to 6 weeks following completion of the program. Data were analyzed using thematic analysis.

**Results:**

Four overarching themes emerged, along with 11 subthemes: (1) Lack of confidence and knowledge, fears surrounding physical ability and symptoms, coupled with issues not addressed as part of the healthcare pathway played a part in transitioning to inactivity; (2) Improvements in strength, mental well-being, physical fitness, fatigue, abdominal and joint pain, comorbidities, and self-management strategies were among the reported benefits of exercise participation; (3) Seeing progress, goal setting, enjoyment, and a peer-led program receiving support and advice increased motivation. Whereas work-related tiredness, other commitments, and self-directed exercise were reported as exercise barriers; (4) The intervention design was well received and the journey from start to finish was positively discussed, important considerations for future interventions and implementation strategies.

**Conclusions:**

The study yielded novel perceptions on the transition to inactivity following receiving a diagnosis, physical and psychological benefits accruing from the intervention, and views on program design. Information that will provide an essential step in the development of implementing exercise guidelines into the clinical pathway and supporting individuals with self-management options.

## Graphical Abstract

**Graphical Abstract:**
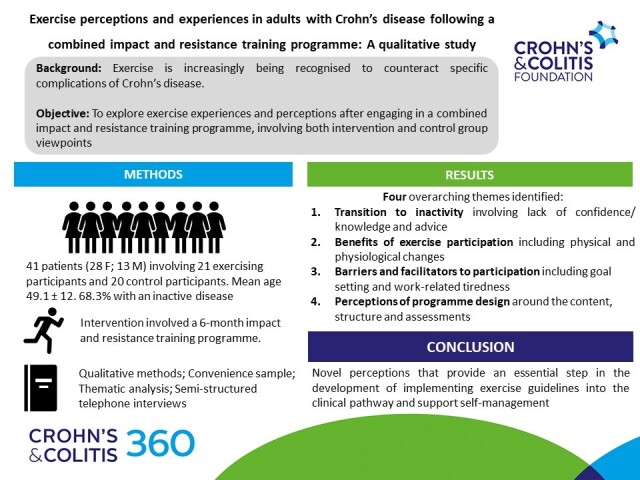

## Introduction

Crohn’s disease (CD), a type of inflammatory bowel disease, is an immunologically mediated idiopathic chronic inflammatory disorder of the gastrointestinal tract, typically occurring at ages 15–30.^1,2^ Characterized by a cyclical nature alternating between remission and relapsing states, patients often suffer from abdominal pain, weight loss, malnutrition, blood loss, and diarrhea.^3^ As a result of disease-specific proinflammatory cytokines, glucocorticoid usage, and malnutrition more than one-third are affected by extraintestinal manifestations involving the musculoskeletal, dermatological, pulmonary, cardiovascular, ocular, and renal systems,^4–8^ which can be just as debilitating as the primary disease.

With no known cure, disease management and treatment aims focus on mucosal healing, deep remission, and preventing the need for surgery.^9^ While effective, most pharmaceutical compounds elicit a wide range of sides effects that may reduce quality of life (QOL) and medication adherence leading to relapse, hospitalization, and increased healthcare costs.^10,11^ Studies have shown corroborating evidence that exercise is a safe nonpharmacological option for people with CD, with reported benefits including improvements in muscle function, psychological well-being, QOL, fatigue, and bone density.^7,12^ However, cross-sectional evidence from a UK-based survey identified that 83% of adults with inflammatory bowel disease were not meeting recommended physical activity guidelines of 150 minutes of moderate aerobic physical activity a week,^13^ similar findings that have been demonstrated worldwide.^14–16^ Strategies are warranted to address common barriers to facilitate increased levels of engagement.

We recently reported the results of a randomized controlled trial (RCT) PROTECT (*PRO*gressive resistance *T*raining *E*xercise and *C*rohn’s disease *T*rial), which investigated the effect of a 6-month combined impact and resistance training program on bone density and muscular function in adults with inactive to mildly active CD.^17^ The program consisted of 3 exercise sessions per week, lasting approximately 60 minutes, for 26 weeks. Twelve tapered supervised sessions were offered on a group or 1-1 basis with an exercise facilitator with the remaining 66 completed unsupervised in a home-based setting. Of the 1716 exercise sessions that were prescribed 1057 (62%) were completed, corresponding to 2 sessions each a week which is consistent with physical activity guidelines for the general adult population. This was also accompanied by improvements in lumbar spine bone density, muscular function, fatigue severity and QOL following the study’s conclusion.

To date no studies have considered a qualitative exploration of exercise experiences and perceptions in adults with CD after an exercise program. Therefore, the aim of this qualitative study was to investigate the views and values of adults with CD after participation in a 6-month combined impact and resistance training program, from both intervention and control group viewpoints.^17^ Improving our understanding of exercise perceptions and personal experiences can aid in the development of supporting individuals with self-management options and inform future healthcare implementation strategies to enhance patient pathways of care.

## Materials and Methods

### Study Design and Sampling

This study used a qualitative research method involving semistructured interviews to explore exercise perceptions and experiences of participants who had taken part in a 6-month exercise program, regardless of group allocation and compliance rates. Recruitment was from a large Hospital Trust in Northern England: Newcastle upon Tyne Hospitals NHS Foundation Trust. Details of the RCT have been previously published elsewhere.^17^ A convenience sample of 43 participants were invited to take part in a qualitative interview. Two participants, one from each group, declined participation and 41 participants were interviewed within 6 weeks of completing the program. A time of within 6 weeks was applied based on previous qualitative research between receipt of an exercise intervention and interviews to understand experiences.^18–20^ This allowed a balance between experiences being “fresh” and allowing enough time to reflect, whilst identifying any unanticipated benefits/harms which may have emerged several weeks later.

### Data Collection

Forty-one participants, consisting of 21 exercising participants and 20 control participants, took part in individual semistructured telephone interviews (20–50 minute duration). All interviews were undertaken by a member of the trial team (K.J.) who had recruited, completed study assessments, and delivered the exercise intervention. Thus, a rapport was established between all participants and the interviewer prior to conducting the interview. Data were collected using an interview guide consisting of open-ended questions on CD history, research participation and specifically for the exercise group: intervention experience, outcomes, and acceptability (Supplementary Material 1), based on previous literature.^21^ All participants were briefed about the purpose of the discussion to elicit views and experiences of engaging in the program. All telephone interviews were audio recorded and later transcribed verbatim. Participant identification numbers were used throughout to maintain anonymity.

### Ethics

Ethical approval was granted by NHS Newcastle and North Tyneside Research Ethics Committee (Ref: 17/NE/0308) and Northumbria University Ethics Committee (Ref: 656). Written informed consent was obtained prior to each interview.

### Data Analysis

Data were transcribed verbatim and analyzed using Framework analysis.^22,23^ This type of thematic analysis uses interrelated steps to assist the management of qualitative data and analysis.^23^ The 2 researchers (K.J. and J.N.) familiarized themselves with the data and coded the first 6 transcripts independently to develop a thematic framework. The 2 researchers then met to compare and discuss the codes and refine the thematic framework. This process ensured 2 perspectives from the data given the different researcher backgrounds of exercise physiologist and physiotherapist, respectively. The remaining transcripts were split between the researchers and thematic framework applied; however, this framework was flexible to allow new codes to emerge. The 2 researchers had frequent meetings to discuss any new codes, and the earlier transcripts were all reviewed when new codes were added to the framework. Any disagreements were resolved through consensus of a third author (G.A.T. or K.B.).

Following this, charts of the data were developed and made into a Framework Matrix. This allowed the researchers to explore the full dataset and facilitate the next stage of data interpretation. Mapping similarities, differences, and connections in the data was carried out by the 2 researchers at this charting stage. Themes were then developed and refined through frequent meetings to allow for reflection and discussion and to ensure both researchers perspectives were considered and captured within the data analysis. The final themes were discussed with the coauthors (G.A.T. and K.B.) and then further refined.

## Results

Participant characteristics are presented in Table 1 and Supplementary Material 2, respectively. Of these participants, 68.3% were female (*n* = 28) with a mean age of 49 ± 12 years 43.9% were employed full time and all were of White ethnicity. The median duration since diagnosis was 204 months (IQR 60–383) and most had an inactive disease (68.3%).

**Table 1.** Participant characteristics.

	All (*n* = 41)	Intervention (*n* = 21)
Age, mean (SD), years	49.1 ± 12	47.5 ± 11
Gender, *n* %		
Female	28 (68.3)	14 (66.7)
Male	13 (31.7)	7 (33.3)
White ethnicity, *n* (%)	41 (100)	21 (100)
Employment status, *n* (%)		
Employed full-time	18 (43.9)	10 (47.6)
Self-employed	5 (12.2)	4 (19.1)
Unemployed	6 (14.6)	2 (9.5)
Retired	8 (19.5)	5 (23.8)
Age at diagnosis, median (IQR), years	31 (23–37)	31 (22–36)
Duration of diagnosis, median (IQR), months	204 (60–383)	216 (108–312)
Baseline disease activity, median (IQR)		
Fecal calprotectin, µg g^−1^	52 (41–98)	45 (36–54)
CDAI	101 (65–158)	98 (50–151)
Week 26 disease activity, median (IQR)		
Fecal calprotectin (FC), µg g^−1^^a^	141 (75–200)	79 (48–113)
FC: number of participants >250 µg g^−1^^a^	4	1
CDAI^b^	95 (40–157)	75 (35–96)
CDAI: numbers of participants >220^b^	2	0
Baseline CDAI activity status, *n* (%)		
Inactive (<150)	28 (68.3)	14 (66.7)
Mildly active (=150–219)	13 (31.7)	7 (33.3)
Week 26 CDAI activity status, *n* (%)^b^		
Inactive (<150)	28 (71.8)	17 (85)
Mildly active (=150–219)	11 (28.2)	3 (15)
Surgical history^c^		
Stoma	6	4
Resection	16	7

Abbreviations: CDAI, Crohn’s Disease Activity Index; IQR, interquartile range.

^a^Based on 34 participants (*n* = 17 intervention).

^b^Based on 39 participants (*n* = 20 intervention).

^c^Multiple answers possible.

**Figure 1.**
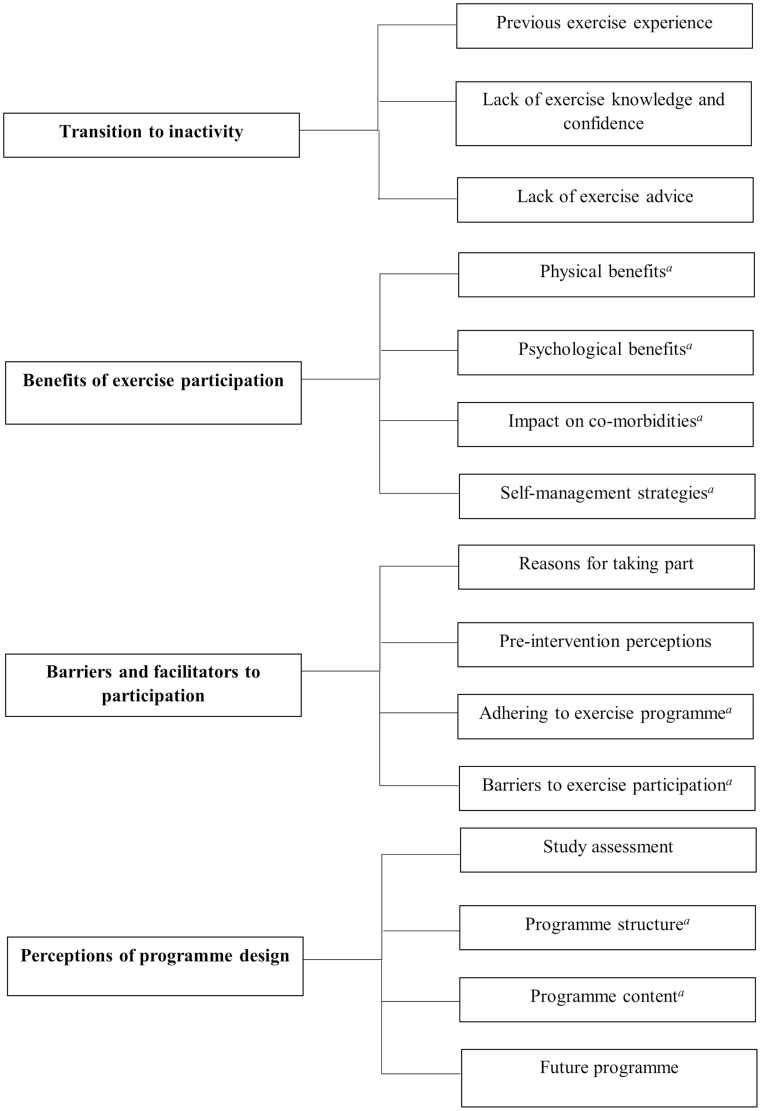
Themes and subthemes from participant views. ^a^Responses from exercise participants only (*n* = 21).

Themes and subthemes applicable to each group are presented in Figure 1. Four overarching themes were identified through thematic data analysis: (1) transition to inactivity; (2) benefits of exercise participation; (3) barriers and facilitators to participation; (4) perceptions of program design. Direct quotes with reference to the participants identification number are presented to illustrate key and subthemes, further quotes are provided in Supplementary Material 3.

### Transition to Inactivity

#### Previous exercise experience

Many participants recollected how exercise played a part in their life before diagnosis, for example cycling, running, swimming, and football. However, following receiving a diagnosis or experiencing a flare up they never returned to exercise.

I used to run a lot before I got diagnosed and after getting that diagnosis my fitness er it just disappeared…running with my diagnosis just didn’t work I couldn’t get out far out the street without feeling like I needed to go to the toilet so then I got nervous about doing other activities especially in the gym so I just stopped (F008)

#### Lack of exercise knowledge and confidence

Despite knowing that exercise was beneficial and a consensus that they should do it, many were unsure what they were able to do, what was safe to do or how to start exercising again after experiencing prolonged periods of inactivity. In most interviews, participants expressed they had initial concerns to engage in an exercise intervention citing reasons around ability, perceived negative effects on abdominal pain, bowel habits, fatigue, and mental well-being. Some also perceived to start exercising again would require going to a gym setting, which made them uneasy as they felt judged at their lack of physical fitness.

I haven’t I’ve not tried to do it because I’ve never really known what to do (R038)

I did join the gym, but they are all so fit [laughs] I went once and actually felt like crying when I came out I just felt so out of place you know, I was unfit, I felt like my condition was holding me back and I just couldn’t do what everyone else seemed to be able to do (R031)

The opposite was seen in some who were able to continue to exercise following a diagnosis and incorporate the likes of Pilates, golf and yoga into their lifestyle. Many expressed they were disheartened about their ability when undertaking the same sporting activities prior to receiving a diagnosis or experiencing a flare up which made the activity less enjoyable. Some also had the idea that they needed to avoid certain exercises because of the condition such as abdominal work and stuck with exercises they were comfortable doing.

I have had surgery and my muscles are weaker and I wasn’t really sure whether I could do any abdominal work and whether if I did do something it would make me need to go to the toilet (F008)

I think I’ve not really explored any types of exercise I used to only do what I knew I was comfortable doing which probably doesn’t help as you don’t like you don’t push yourself (R046)

#### Lack of exercise advice

There was a consensus that exercise advice was not discussed with healthcare professionals. This played a part in participants inactivity with not knowing what is safe to do from an authoritative role they trusted and doubts over whether they should exercise at all if it is not discussed. Interestingly, some participants discussed the idea of when the most appropriate time for exercise advice should be given to the individual, reflecting to early days in their diagnosis. There was a consensus that this should depend largely on how long the individual have been diagnosed with many recognizing the diagnosis itself was a lot to take in and been given additional information on exercise would have been too much.

I have mentioned to my GP previously about doing exercise but they didn’t really know what I should be doing and whether it would be okay after I’ve just had surgery so I never bothered (R013)

I just think back to when I was diagnosed and it was a lot to take in and considering anything else [exercise] would have been extremely overwhelming. It was coming to terms with how it was going to impact my life so I personally would have found it too much to do (R037)

### Benefits of Exercise Participation

#### Physical benefits

Participants universally reported an increase in strength and improved fitness, that translated into functional improvements such as being able to walk further, posture, sleep better, help with weight loss, improve appetite, and encourage to eat better. For some, this improved health and fitness subsequently created opportunities to engage in social activities with family. Two participants, both within 4 months of surgery, also suggested that the exercise program helped them recover quicker.

I feel generally fitter in myself I noticed I’m not getting out breath doing everyday activities and my grandkids love it as I’m able to run with them in the part and kick the football back (R046)

I feel like the Michelin Man (R015)

…after my operation I feel like it [exercise programme] was something that helped me bounce back quicker. I know there’s no running back from surgery I get that but I do feel like this helped me get to a stage in my life I didn’t think I’d get back to for a lot longer (F001)

I wasn’t really sure what it would do with me being, well having surgery months before hand but its been just what I needed to get back on my feet and just give me my own independence back (R013)

Some participants explained how they noticed a reduction in the severity and frequency of abdominal and joint pain experienced post intervention, others reported no change or worsening in symptoms. Despite participants expressing their initial concerns for potential worsening of symptoms, particularly when concerning fatigue, they were pleasantly surprised when this was not the case. In some cases, participants expressed they felt more energized, which meant more freedom for people to engage in activities that they would like to do.

I definitely think it’s helped with my fatigue, I’ve tried iron and dietary stuff to try and give me more energy [pause 2 seconds]…but the exercise I found, well, it gives me a boost and I find myself doing more with my day (R039)

…over the moon as now I know what I can do symptom free, what more do you want (R030)

#### Psychological benefits

Participation in the exercise program also led to psychological benefits. Within the interviews this area had a lot of coverage from participants. Improved well-being, life changing and feeling the best people have felt in a long time were each commented upon. Whilst feeling these changes in themselves, participants also commented on others noting a change in them and feeling positive regarding this. The exercise program increased confidence in appearance, but also in exercising again in knowing that it would not flare up their condition. Sense of achievement or feeling proud for completing the program, or for hitting a goal was also noted.

I feel positive, I feel overwhelmed to be honest with you, really with how good I feel, never thought I’d ever feel like this again after being diagnosed (R032)

…one of the best things I did and I’ve learnt so much about myself and yes it’s just been a very erm good and insightful for me and I feel hugely proud of myself for doing and giving everything in to the programme (F023)

…thrilled with the results and my muscles as I was with the new car. But just to make progress in any direction would be a help and I just wondered if I was able to do it so I got a great sense of achievement. I really enjoyed doing it (R015)

#### Impact on comorbidities

The presence of other long-term chronic conditions, often linked with CD were discussed. These included ankylosing spondylitis, depression, anxiety, chronic obstructive pulmonary disease (COPD), joint aches, and arthritis. Although the exercise intervention was designed specifically to target bone density and muscular function, it had transferrable benefits on other symptoms experienced.

I mean at one time when I was doing exercises before this my knees were terrible, the bottom of my back has always been terrible yano. Em, my shoulders have always ached, but I just don’t have hardly any of that sort of pain now (R015)

I would think it [exercise programme] has because I’ve not had any pain in them [joints], especially my fingers, my fingers sometimes really hurt with my AS… (R039)

I’ve noticed I haven’t had as many erm, twinges like aches in it [Knee] like I did before the programme (F015)

#### Self-management strategies

There was a general consensus that the exercise intervention offered new insights into the condition. Many felt that having the knowledge, including what, when, and how to perform the exercises could enable them to help themselves, take control, and even held themselves accountable to see change. Participation in the exercise program also led to an increased knowledge of capabilities after experiencing a flare up, surgery or going a prolonged time without any physical activity.

I knew that Crohn’s caused a lot of other problems but I have, well I know more about what I can do to manage stuff myself (R027)

This programme has definitely been something that has encouraged me to get my life back on track to eat better, to concentrate on the things I like doing and just taking charge a little bit more, stronger control (F001)

it’s also interesting to see how your condition is after the 6 months because you don’t know what’s going to happen and if it does flare up. Its good now I have that knowledge to know that I can do what we did, it not flared me up and it’s been beneficial, something that I want to keep up (R002)

For some the intervention changed how they viewed their condition with realisations of acceptance, understanding, and coping. However, for some the program did not change how they saw their condition, this was particularly apparent in participants with a longer disease duration.

I mean I’ve been diagnosed along time and there’s been new stuff about it come out all the time, so nothing surprises me with it (R009)

I see it more positively, I think knowing that although it may get the better of me sometimes I can do stuff and it won’t stop me, so yeah I think it [exercise programme] has made me realise that I’m someone that has Crohn’s but that’s not the end of the world and you can still enjoy yourself even if times are hard sometimes (F023)

It has [exercise programme] helped paint a bigger picture of the condition for me, think cos I was diagnosed really young I never really knew what was going on, I had to get on, [pause 3 seconds] I didn’t know any different but I’m still learning about it and that’s cool (R013)

### Barriers and Facilitators to Participation

#### Reasons for taking part

Participants were overwhelmingly excited about the prospect of the study to help family members, friends, or others with the condition and the impact the results may have on their future generations and management options available. The relationship between healthcare professionals and the individual also played an important part when considering their decision to participate, such as trusting their knowledge and wanting to give something back.

There’s not a lot out there in terms of Crohn’s so anything that you can do to help in terms of Crohn’s in others, even if it’s not my sort of well-being I’m interested in being a part of it for others (F007)

I want to know more not that it might help me in my lifetime but it might help my kids and my grandkids (R021)

Motivation for many participants was also underpinned by the goal of wanting to improve knowledge of the condition and exercise because of the potential it offered. Many wanted to discover whether they could exercise without impacting symptoms and establish physical fitness abilities and boundaries in a safe environment. Exploring alternatives to their current treatments was also a motivation for to participation, with participants recognizing the importance of their participation in advancing knowledge around complementary therapies. For some participation was strongly influenced by past experience participating in exercise.

Selfishly in a way I guess I just wanted to know more about the condition and see what the study showed whether we could look at other options for the disease. I think 18 years of medications and surgeries is a lot and I’m starting to get to that stage where I’m running out of options, so the more options, whether that is targeted directly at the disease or complications with it then I’m more than happy to give blood, samples, heck I’d even have a colonoscopy [laughs] (F021)

#### Pre-intervention perceptions

Many participants had preconceptions about the exercise program including the intensity, setting, and type. Although none of the participants had previous experience of using resistance bands, and very few had experience of strength training participants expressed how excited they were to try something new or something they had not heard about previously. A few participants discussed how they thought using the resistance bands would be easier than weights and that they would find the program quite easy which to their surprise was not the case. A few female participants expressed they had previously avoided strength training for fears this would make them look too manly, or too muscular as they were unsure what they were doing. Some participants expected the exercise sessions to be conducted in a gym or gym class not specifically in a research facility. However, noted they were pleased to see where the research was being carried out and found the “lab setting interesting.”

I was excited to try something I never had before to see what I could get out of it (F009)

I’d never done strength training before just because I always thought like erm I was too weak or that id end up looking manly (R002)

#### Adhering to exercise program

Objective improvements such as seeing physical or psychological progress was strong motivator for adhering to the program and continuing to attend sessions. Enjoyment was also a strong motivator, with some participants discussing how they continued with the program despite being on holiday, taking their resistance band with them. Many also noted they were continuing to engage with exercise after the program had finished, despite this question not being asked, stating they wanted the intervention to go on longer due to enjoyment. Some felt that if they gave up it would have taken that opportunity away from somebody else. Whilst others felt was their responsibility to complete the program to enhance scientific knowledge.

I’ve done it to the nth degree I mean you know I’ve done it all over the world when we’ve been to Brazil and Portugal and Peru in the past 6 months, the people at the hotel probably thought I was crazy [laughs] but I can feel the difference and deserve the results that I got [laughs] (R015)

…this kind of thing is going to help someone else further down the line if they’ve diagnosed with Crohn’s, or helping exercise to get the energy up. I just had that in the back of me mind… (F019)

The program itself was peer led. Participants valued this, in particular the rapport built up with the facilitator through a shared experience and understanding. People reported they may have felt self-conscious regarding having to leave the sessions to use the toilet, however they noted as this was peer led, that this removed a sense of feeling judged if they were to leave a number of times. Alongside this if individuals were unwell, being peer led made participants feel the instructor would understand. The facilitator provided a key source of motivation for participants. Some participants did not often discuss living with Crohn’s with anyone, however due to the peer-led nature of the program felt able to discuss the impact this was having and any concerns or queries regarding exercise.

…be in an environment where someone understood the condition (R046)

The researcher doing the assessments I felt was very friendly, motivating, informative, erm very knowledgeable about the condition which I felt was a real benefit as I don’t think I would have enjoyed attending the sessions with someone who didn’t understand the disease (R002)

#### Barriers to exercise participation

Exercising at home was agreed by the majority as more challenging than attending supervised sessions due to holidays, childcare and work commitments and work-related tiredness causing struggles to find the motivation to put their exercise intentions into action. However, for some a home-based program was preferred as it allowed the flexibility to fit in with their lifestyle and other commitments. For others it was knowing they were in safe environment, where the toilets were and not being judged by their ability as experienced previously in a gym environment.

… I’ve got a couple of little children and domestically and everything like that. I’m working full time and erm, weekends we’re doing stuff and it’s just finding the time to do it (F022)

I stood in front of the telly watched the programme and did my exercising. So, yeah, yeah, the fact you could do it at home or anywhere at any time of the day was great. (R012)

Being able to do them at home after having those sessions was great [pause 2 seconds] made me feel more at ease like you weren’t being judged for not being able to keep up which I thought at the gym (R002)

### Perceptions of Program Design

#### Study assessments

An overarching finding was individuals’ resilience to assessments. Participants discussed being used to assessments due to living with their condition, therefore these were not perceived as an issue as part of the study. An example often discussed was providing a stool sample. Many of the assessments included within the study, individuals discussed having had a number of times previously. Assessments were often combined with routine visits at the hospital, which was highlighted as beneficial.

I felt they were all fine, nothing that was out of the ordinary, it was straight forward I knew the hospital and where I was going so it was very at ease with what was going on (F009)

Didn’t bother me at all, I’m just perfectly used to all that sort of thing, they’re just routine to me (R015)

There were some additional assessments as part of the study, which included a bone scan and muscular testing. People were often interested in the additional tests process and findings. In particular, individuals were pleased to have the bone scan and to understand the results of this. Many participants had not experienced this assessment previously.

I actually thought it was fun and was really useful to have you know, I’d never have been given a chance otherwise to have my bones tested or my muscles like that so yeah fantastic (R030)

#### Program structure

The journey through the program was positively discussed by participants. Prior information including the participant information sheet and the exercise booklet were found to be very helpful. Initially individuals felt there was a lot to take in however these materials helped with understanding. Participants also commented on the importance of understanding the layout and facilities, knowing toilets were near and accessible was important and being shown this at the start was commented as helping participants to feel at ease.

You could take your book and your resistance bands and even you could have quite a number of holidays [laughs]. You could take them on holiday with you and you could do it anywhere. They just slip into your suitcase so that was the beauty of it (R015)

Initially, the length of the program was viewed to be quite long, however, once the program commenced participants often commented how quickly it went and commented on the length positively. The length of the exercise sessions themselves allowed people to build up gradually over the course of the session and viewed this as being about right in length. Travel presented no issues for participants.

At the start I thought it was doing to be quite long, but then when you said we were at 3 months it just flew by it really did and I think at that stage I was really seeing what my potential was and wanted to do more so 6 months was just right I think for what the goals of the research are (R024)

Many individuals had initial concerns that the intensity of the exercise program may be too difficult. Alongside this, there were concerns regarding an adverse effect of exercise on symptoms. A balance was needed as participants wanted to be mindful of their symptoms however at the same time, wanted to carry out exercise at an intensity pace they were comfortable with. The gradual pacing of the exercise program played an important role as it allowed participants to have this balance.

I think obviously at first you think it’s too much, but that’s because your starting from nothing almost. I mean you know what I was like I came back for the second session so it didn’t put me off [laughs] but it was hard and it, I was really shocked to see how far down I must have been, but I don’t think you can make it too easy because then like you just don’t really see any benefits (R038)

#### Program content

The majority of participants preferred using the resistance bands than gym equipment, as they were adaptable in length, resistance and easy to carry to and from location. The different band colors (4 in total) allowed individuals to see their progress, which gave participants a sense of achievement and something to work towards. Jumping as part of the program was often mentioned by participants. Initially most were surprised how difficult they found this having not done it before, however as the program progressed individuals found this easier and saw their fitness levels improve.

…I liked that the bands and the exercises could be made easier or harder and like you went through the colours erm, so yeah when you said that in the first session it was my challenge to myself to get to the blue band [laughs]. I got to the green but I’m happy with that and what I’ve achieved (R009)

Universally participants would recommend the exercise program to others with and without the condition, highlighting both the physical and psychological impact. Availability of exercise via the NHS was encouraged by most participants with positive views of this type of program being rolled out. Many addressed that exercise was available via the NHS for other health conditions however were disappointed this was not offered for CD, nevertheless nearly all participants would be willing to pay for exercise sessions.

It [exercise] is available for other conditions so why not Crohns. It gets frustrating when Crohn’s isn’t even thought about, if it causes, which is does, these problems then we should have the information to be able to help ourselves, I think that should be an option for us instead of just the surgery or the erm medications (R032)

I would pay for something like that that you are doing it the right way, your targeting areas that the like the Crohn’s is affecting so it’s going to help now and in the future… (F019)

#### Future exercise intervention recommendations

For some individuals more variation in the program was discussed as a future improvement. One suggestion included changing the exercises performed part way through the program, particularly so it did not feel as repetitive when supervised sessions tapered off. Some participants discussed that they would like more supervised sessions to provide motivation, or so it helped them establish more of a structure with their lifestyle. Whilst participants enjoyed the sessions as they were, it was felt group sessions, or a buddy system could be trialed to share experiences with other individuals participating in the program. Group sessions were offered, however due to flexibility of the program and availability of participants this was not always an option.

I mean the buddy system. Then, then I think I’ve mentioned that before, but like, you know, you’ve got to set up some kind of WhatsApp group as well with everyone as it would be good to meet others with the condition and share exercise tips (F001)

I think me personally erm, just maybe more sessions, longer, if it went on longer as well and it just flew by, cannot believe its gone this quick. So would be good to know what to do after it, I’ll keep it up, but just be good to know other things that I basically do (F015)

You’ll be pleased to know Katherine I’ve carried on doing the programme I’ve done my jumps and done my reps and it’s been what 3, 4 weeks since it stopped (R015)

## Discussion

The purpose of this research was to investigate the perceptions and experiences of both intervention and control group participants after participating in a 6-month combined impact and resistance training program. Our findings provide novel insights from both intervention and control group viewpoints into how participants transitioned into inactivity, the benefits of exercise yielded from the intervention, views on challenges and facilitators to participation and thoughts on the study and intervention design features.

Following receiving a diagnosis or experiencing a flare up many participants developed a lack of confidence to exercise, uncertainty around how to safely engage in exercise and fears surrounding physical ability or exacerbation of symptoms. Coupled with issues not addressed as part of the healthcare pathway played a part in transitioning to inactivity (Theme 1). Fear of exacerbating symptoms such as worsening of abdominal pain or fatigue have been widely reported as obstructing factors to exercise participation in people with CD.^16,24,25^ Participants valued the gradual pacing of the exercise program to avoid feeling fatigued and overworking the abdominal muscles. Our findings also highlighted the importance of empowerment and building confidence to diminish fears such as not knowing how their condition was going to respond to exercise or what their physical abilities were which were aided by the gradual pacing with support of expert advice.

Participants also felt the lack of exercise advice given by healthcare professionals played a part in them questioning whether they should exercise, if it was safe to do and what they should do for their condition. The lack of advice from healthcare professionals could be due to the lack of specific guidelines surrounding the type, frequency, intensity, and duration of exercise in CD. Although preliminary findings suggest that exercise is feasible, safe and may have beneficial effects on a diverse range of health outcomes,^7,12,26^ a substantially larger body of evidence is needed to identify the optimal exercise prescription in developing guidelines to enable recommendations and optimize health outcomes. Although participants universally agreed exercise advice should be given by healthcare professionals or provided by the NHS, interestingly the timing of this advice was raised as a concern, particularly around diagnosis. Further research exploring individuals’ perceptions around implementing exercise guidelines and advice is warranted to establish when it would be most effective to integrate into the clinical pathway.

The reported improvements in strength and physical capacity, consistent with previous reports,^7,12,26^ created opportunities for participants to engage in more social and physical activities with family (Theme 2). Of particular interest in this study were the benefits participants reported regarding comorbidities, abdominal and joint pain, something not readily collected or often quantifiable, thus highlighting the importance of qualitative investigations in identifying what exercise benefits are most valued by people with CD. To our knowledge, improvements in comorbidities, abdominal and joint pain following an exercise intervention in adults with CD have not been described elsewhere. Improvements in joint pain and comorbidities such as ankylosing spondylitis and COPD may not be as surprising given exercise is a core treatment in NICE guidelines,^27,28^ as it is for managing joint conditions associated with CD such as osteoarthritis^29^ and rheumatoid arthritis.^30^

There is a growing body of evidence that structured exercise programs rather than short bouts improve gut barrier integrity, which may explain the perceived improvements in abdominal pain.^31,32^ However, despite short bouts of exercise negatively impacting gastrointestinal symptoms and gut permeability^33–35^ this does not appear to be the case in individuals with CD who after acute exercise saw no changes in gastrointestinal symptoms, gut permeability, or lipid peroxidation.^36,37^ More research exploring the mechanisms between gut symptomatology and exercise of different intensities, types, durations, and rest intervals to assess promotion of gut integrity in this population is warranted.

Aside from physiological improvements, the role the program had for enhancing psychological well-being was apparent. Previous findings have identified improvements in QOL, anxiety, and depression after an exercise intervention.^7,12,26^ However, within this study, participants placed greater value on accomplishments, making better lifestyle changes, having more confidence in their appearance, and feeling proud which should not be overlooked. Participants agreed the program increased their knowledge of exercise, however understanding and seeing their condition was more apparent in those who were more recently diagnosed than those with a longer disease duration. However, both, universally agreed their newly found exercise knowledge would impact how they would manage their condition going forward. Self-management is extremely important in long-term conditions, aiming to empower and create a more sustainable way of living.

Altruistic motives such as helping others and recognizing their participation in scientific advancement remain a strong motivator for research participation (Theme 3).^38,39^ Personal benefit was also influential, with some individuals hoping it would further their knowledge of the condition and exercise and would lead to alternative treatment options. Trusting the opinions of healthcare professionals also played a part in considering participation, which is often reported as a barrier to research participation.^38,40^ Therefore, the impression of healthcare professionals and the information they provide to CD participants is crucial for trial recruitment but could also be essential in promoting exercise in routine practice.

Several motivational factors that encouraged exercise participation were identified such as setting and reaching goals, enjoyment and wanting to continue to contribute to science. Whilst initial motivation to undertake an exercise program may be present, considerations surrounding continuing to exercise to contribute to science may result in participants feeling pressured to deliver or guilt. Feelings of guilt and pressure to achieve can affect motivation and negatively impact long-term exercise participation.^41^ One significant aspect highlighted in this study was the importance of physical and psychological quantifiable changes and tangible goals to maintain motivation. Enjoyment was also reported as an important facilitator, and a vital predictor of intending to continue exercising and adherence, relevant outcomes capable of promoting behavioral change.^42,43^

A peer-led program was also highlighted as an important facilitator to exercise participation, a finding not previously reported within this population. Participants valued this, building up a rapport and trust through a shared experience and understanding. Systematic review evidence demonstrated that peer-led health interventions in both older adults^44^ and adolescents^45^ positively impacted health behavior and produced impactful changes. A greater emphasis on peer-led programs is warranted for future interventions. Conversely, struggles to find motivation as a result from work demands or family obligations were identified as barriers to exercise participation. Although strategies such as goal setting and self-monitoring were used, systematic review evidence highlights the importance of engaging with social support networks from family and friends and through group-based intervention delivery.^46,47^ Strategies to engage these support structures should be built into future programs.

Resilience in inflammatory bowel disease has previously been reported,^48,49^ but there were some new insights into how resilient participants were to study assessments, particularly stool tests (Theme 4). Coordinating these assessments with routine appointments was seen as important, a finding not previously described elsewhere, meant reduced burden for the participant and any unnecessary stress and worry associated with travel. The exercise program structure and content, despite preconceptions surrounding the intensity, setting, and type of exercise, were well received from start to finish. The intervention materials were positively discussed, as was the proximity of toilet facilities which reassured participants that they were in a safe environment. In cross-sectional surveys the lack of toilet access has frequently been acknowledged as a limiting factor to exercise.^24,25^ Thus, preferences related to location of exercise sessions and assessments are important considerations for future interventions.

In agreement with previous findings,^50^ home exercising was hard to sustain for some participants, despite their positive exercise experiences. In contrast to this, there were some participants who preferred a home-based program, finding this fitted better with their lifestyle, led to no fitness judgments, and made them feel at ease being close to toilet facilities. One of the future recommendations by participants was to have more supervised sessions to help maintain motivation and structure, whilst this may not always be possible engagement with participants is important to elicit behavioral change^46^ and future research should explore the different ways to communicate with participants (face to face, video conferencing, a support webpage).

### Strengths and Limitations

The trustworthiness of this research has been enhanced in several ways. Two independent coders conducted data analysis, enhancing the credibility of the research. These 2 researchers had different backgrounds of exercise science and physiotherapy. This allowed different perspectives when analyzing the data and for open reflection and discussion during theme development. The wider research team allowed further reflection on researcher influences and theme development with peer debrief meetings. Another strength of the study is the richness of information in the interview data, which may be as a result of including viewpoints from both the intervention and control groups. By including multiple perspectives, including those of the control group allowed for a broader understanding, provided valuable insights, and enhanced transferability of the complex aspects surrounding views on exercise in addition to their unique perspectives on the intervention which they did not engage in.

An important limitation of this study was the inclusion of only participants with an inactive to mildly active disease who were involved in the RCT and qualitative investigation. Future research exploring the views of exercise participation is needed to understand any specific and individual challenges posed from individuals with a more severely active disease. It is also possible that views may not be representative of the CD population as participants were recruited from a single center. It is also plausible given that participants recruited to this study had volunteered for an exercise intervention study that they were motivated to engage in exercise than the broader population. Similarly, all participants in the RCT and therefore interviews where of a White British ethnicity. Caution should be applied when generalizing these findings. Future studies may wish to focus on a larger more representative sample including different cultural, social, education, and environmental backgrounds. Lastly, participants had a prior relationship with the interviewer who completed study assessments and delivered the exercise intervention. It is possible that this prior rapport may have influenced the responses given and their experience of the intervention.

## Conclusions

This qualitative research provides a detailed understanding of individuals motivations, attitudes, beliefs, and perceptions surrounding exercise and participation in an exercise program, from both intervention and control group viewpoints. We found participants transitioned to inactivity following receiving their diagnosis, coupled with lack of confidence and knowledge to exercise, fears surrounding physical ability, perceived negative effect on symptoms and lack of exercise advice from clinical settings. A gradual paced peer-led program where support and advice were received and seeing physical and psychological progress were highly valued. In contrast, struggles to find the motivation because of work and family obligations and self-directed exercise were reported as barriers. These findings provide aid in the development of supporting individuals with self-management options and inform future healthcare implementation strategies to enhance patient pathways of care.

## Supplementary Material

otad019_suppl_Supplementary_Material

## Data Availability

All data are available from the corresponding author on reasonable request.

## References

[1] Sartor RB. Mechanisms of disease: pathogenesis of Crohn’s disease and ulcerative colitis. Nat Clin Pract Gastroenterol Hepatol. 2006;3(7):390–407. 16819502 10.1038/ncpgasthep0528

[2] Oliveira SB , Monteiro IM. Diagnosis and management of inflammatory bowel disease in children. BMJ. 2017;357(31):j2083. 28566467 10.1136/bmj.j2083 PMC6888256

[3] Abraham C , Cho JH. Inflammatory bowel disease. N Engl J Med. 2009;361(21):2066–2078. 19923578 10.1056/NEJMra0804647 PMC3491806

[4] Repiso A , Alcántara M, Muñoz-Rosas C, et al. Extraintestinal manifestations of Crohn’s disease: prevalence and related factors. Rev Esp Enferm Dig. 2006;98(7):510–517. 17022700 10.4321/s1130-01082006000700004

[5] Miznerova E , Hlavaty T, Koller T, et al. The prevalence and risk factors for osteoporosis in patients with inflammatory bowel disease. Bratisl Lek Listy. 2013;114(8):439–445. 23944617 10.4149/bll_2013_092

[6] Vavricka SR , Schoepfer A, Scharl M, Lakatos Peter L, Navarini A, Rogler G. Extraintestinal manifestations of inflammatory bowel disease. Inflamm Bowel Dis. 2015;21(8):1982–1992. 26154136 10.1097/MIB.0000000000000392 PMC4511685

[7] Narula N , Fedorak RN. Exercise and inflammatory bowel disease. Can J Gastroenterol. 2008;22(5):497–504. 18478136 10.1155/2008/785953 PMC2660805

[8] Ott C , Schölmerich J. Extraintestinal manifestations and complications in IBD. Nat Rev Gastroenterol Hepatol. 2013;10(10):585–595. 23835489 10.1038/nrgastro.2013.117

[9] Gajendran M , Loganathan P, Catinella AP, et al. A comprehensive review and update on Crohn’s disease. Dis Mon. 2018;64(2):20–57. 28826742 10.1016/j.disamonth.2017.07.001

[10] Kane SV , Cohen RD, Aikens JE, Hanauer SB. Prevalence of nonadherence with maintenance mesalamine in quiescent ulcerative colitis. Am J Gastroenterol. 2001;96(10):2929–2933. 11693328 10.1111/j.1572-0241.2001.04683.x

[11] Levine JS , Burakoff R. Extraintestinal manifestations of inflammatory bowel disease. Gastroenterol Hepatol (N Y). 2011;7(4):235–241. 21857821 PMC3127025

[12] Eckert KG , Abbasi-Neureither I, Köppel M, Huber G. Structured physical activity interventions as a complementary therapy for patients with inflammatory bowel disease—a scoping review and practical implications. BMC Gastroenterol. 2019;19(1):115-127. 31266461 10.1186/s12876-019-1034-9 PMC6604412

[13] World Health Organisation. WHO Guidelines on physical activity and sedentary behaviour. 2020. Accessed July 20, 2022. https://apps.who.int/iris/bitstream/handle/10665/336656/9789240015128-eng.pdf

[14] Van Langenberg DR , Papandony MC, Gibson PR. Sleep and physical activity measured by accelerometry in Crohn’s disease. Aliment Pharmacol Ther. 2015;41(10):991–1004. 25783784 10.1111/apt.13160

[15] DeFilippis EM , Tabani S, Warren RU, Christos PJ, Bosworth BP, Scherl EJ. Exercise and self-reported limitations in patients with inflammatory bowel disease. Dig Dis Sci. 2016;61(1):215–220. 26254773 10.1007/s10620-015-3832-4

[16] Fagan G , Osborne H, Schultz M. Physical activity in patients with inflammatory bowel disease: a cross-sectional study. Inflam Intest Dis. 2021;6(2):61–69. 10.1159/000511212 PMC8160568 34124177

[17] Jones K , Baker K, Speight RA, Thompson NP, Tew GA. Randomised clinical trial: combined impact and resistance training in adults with stable Crohn’s disease. Aliment Pharmacol Ther. 2020;52(6):964–975. 33119156 10.1111/apt.16002

[18] Hornbuckle LM , Barroso CS, Rauer A, Jones CS, Winters-Stone KM. “It was just for us”: qualitative evaluation of an exercise intervention for African-American couples. BMC Public Health. 2021;21(1):1–12. 33933048 10.1186/s12889-021-10659-2 PMC8087875

[19] Midtgaard J , Tveterås A, Rørth M, Stelter R, Adamsen L. The impact of supervised exercise intervention on short-term postprogram leisure time physical activity level in cancer patients undergoing chemotherapy: 1- and 3-month follow-up on the body & cancer project. Palliat Support Care. 2006;4(1):25–35. 16889321 10.1017/s1478951506060044

[20] Montgomery CA , Henning KJ, Kantarzhi SR, Kideckel TB, Yang CFM, O’Brien KK. Experiences participating in a community-based exercise programme from the perspective of people living with HIV: a qualitative study. BMJ Open. 2017;7(4):e015861. 10.1136/bmjopen-2017-015861 PMC5387963 28377397

[21] Bottoms L , Leighton D, Carpenter R, et al. Affective and enjoyment responses to 12 weeks of high intensity interval training and moderate continuous training in adults with Crohn’s disease. PLoS One. 2019;14(9):e0222060. 31539378 10.1371/journal.pone.0222060 PMC6754139

[22] Ritchie J , Spencer L. Qualitative data analysis for applied policy research. In: Bryman A, Burgees G, eds. Analyzing Qualitative Data. Routledge; 2002:173–194.

[23] Gale NK , Heath G, Cameron E, Rashid S, Redwood S. Using the framework method for the analysis of qualitative data in multi-disciplinary health research. BMC Med Res Methodol. 2013;13(1):1–8. 24047204 10.1186/1471-2288-13-117 PMC3848812

[24] Chan D , Robbins H, Rogers S, Clark S, Poullis A. Inflammatory bowel disease and exercise: results of a Crohn’s and Colitis UK survey. Frontline Gastroenterol. 2014;5(1):44–48. 28839750 10.1136/flgastro-2013-100339 PMC5369708

[25] Tew GA , Jones K, Mikocka-Walus A. Physical activity habits, limitations, and predictors in people with inflammatory bowel disease: a large cross-sectional online survey. Inflam Bowel Dis. 2016;22(12):2933–2942. 10.1097/MIB.0000000000000962 27824653

[26] Mareschal J , Douissard J, Genton L. Physical activity in inflammatory bowel disease: benefits, challenges and perspectives. Curr Opin Clin Nutr Metab Care. 2022;25(3):159–166. 35238803 10.1097/MCO.0000000000000829

[27] NICE. Chronic obstructive pulmonary disease in adults: quality standard. 2016. Accessed August 17, 2022. https://www.nice.org.uk/guidance/qs10/chapter/quality-statement-4-pulmonary-rehabilitation-for-stable-copd-and-exercise-limitation

[28] NICE. Confirmed ankylosing spondylitis. 2019. Accessed August 17, 2022. https://cks.nice.org.uk/topics/ankylosing-spondylitis/management/confirmed-ankylosing-spondylitis/

[29] NICE. Osteoarthritis: quality standard. 2015. Accessed August 17, 2022. https://www.nice.org.uk/guidance/qs87/chapter/quality-statement-4-exercise

[30] NICE. Rheumatoid arthritis in adults: management. 2020. Accessed August 17, 2022. https://www.nice.org.uk/guidance/ng100/chapter/recommendations

[31] Keirns BH , Koemel NA, Sciarrillo CM, Anderson Kendall L, Emerson SR. Exercise and intestinal permeability: another form of exercise-induced hormesis? Am J Physiol Gastrointest Liver Physiol. 2020;319(4):G512–G518. 32845171 10.1152/ajpgi.00232.2020

[32] Moore JH , Smith KS, Chen D, et al. Exploring the effects of six weeks of resistance training on the fecal microbiome of older adult males: secondary analysis of a Peanut protein supplemented randomized controlled trial. Sports. 2022;10(5):65. 35622473 10.3390/sports10050065 PMC9145250

[33] Ter Steege R , Kolkman J. The pathophysiology and management of gastrointestinal symptoms during physical exercise, and the role of splanchnic blood flow. Aliment Pharmacol Ther. 2012;35(5):516–528. 22229513 10.1111/j.1365-2036.2011.04980.x

[34] Costa R , Snipe R, Kitic C, et al. Systematic review: exercise-induced gastrointestinal syndrome—implications for health and intestinal disease. Aliment Pharmacol Ther. 2017;46(3):246–265. 28589631 10.1111/apt.14157

[35] Hart TL , Townsend JR, Grady NJ, et al. Resistance exercise increases gastrointestinal symptoms, markers of gut permeability, and damage in resistance-trained adults. Med Sci Sports Exerc. 2022;54(10):1761–1770. 35612399 10.1249/MSS.0000000000002967

[36] D’inca R , Varnier M, Mestriner C, et al. Effect of moderate exercise on Crohn’s disease patients in remission. Ital J Gastroenterol Hepatol. 1999;31(3):205–210. 10379481

[37] Ploeger HE , Takken T, de Greef MH, Timmons BW. The effects of acute and chronic exercise on inflammatory markers in children and adults with a chronic inflammatory disease: a systematic review. Exerc Immunol Rev. 2009;15(1):6–41. 19957870

[38] Sheridan R , Martin-Kerry J, Hudson J, Parker A, Bower P, Knapp P. Why do patients take part in research? An overview of systematic reviews of psychosocial barriers and facilitators. Trials. 2020;21(1):1–18. 32164790 10.1186/s13063-020-4197-3 PMC7069042

[39] Naidoo N , Nguyen VT, Ravaud P, et al. The research burden of randomized controlled trial participation: a systematic thematic synthesis of qualitative evidence. BMC Med. 2020;18(1):1–11. 31955710 10.1186/s12916-019-1476-5 PMC6970283

[40] Phelps EE , Tutton E, Griffin X, Baird J. A mixed-methods systematic review of patients’ experience of being invited to participate in surgical randomised controlled trials. Soc Sci Med. 2020;253:112961. 32247942 10.1016/j.socscimed.2020.112961

[41] Teixeira DS , Rodrigues F, Cid L, et al. Enjoyment as a predictor of exercise habit, intention to continue exercising, and exercise frequency: the intensity traits discrepancy moderation role. Front Psychol. 2022;18(13):780059. 10.3389/fpsyg.2022.780059 PMC8894246 35250719

[42] Rodrigues F , Teixeira DS, Neiva HP, Cid L, Monteiro D. The bright and dark sides of motivation as predictors of enjoyment, intention, and exercise persistence. Scand J Med Sci Sports. 2020;30(4):787–800. 31858648 10.1111/sms.13617

[43] Calder AJ , Hargreaves EA, Hodge K. Great expectations: a qualitative analysis of the factors that influence affective forecasts for exercise. Int J Environ Res Public Health. 2020;17(2):551. 31952225 10.3390/ijerph17020551 PMC7013840

[44] Barras L , Neuhaus M, Cyarto EV, Reid N. Effectiveness of peer-led wellbeing interventions in retirement living: a systematic review. Int J Environ Res Public Health. 2021;18(21):11557. 34770069 10.3390/ijerph182111557 PMC8583038

[45] McHale F , Ng K, Taylor S, et al. A systematic literature review of peer-led strategies for promoting physical activity levels of adolescents. Health Educ Behav. 2022;49(1):41–53. 34628981 10.1177/10901981211044988 PMC8892039

[46] Greaves CJ , Sheppard KE, Abraham C, et al. Systematic review of reviews of intervention components associated with increased effectiveness in dietary and physical activity interventions. BMC Public Health. 2011;11(1):1–12. 21333011 10.1186/1471-2458-11-119 PMC3048531

[47] Samdal GB , Eide GE, Barth T, Williams G, Meland E. Effective behaviour change techniques for physical activity and healthy eating in overweight and obese adults; systematic review and meta-regression analyses. Int J Behav Nutr Phys Act. 2017;14(1):1–14. 28351367 10.1186/s12966-017-0494-y PMC5370453

[48] Matini L , Ogden J. A qualitative study of patients’ experience of living with inflammatory bowel disease: a preliminary focus on the notion of adaptation. J Health Psychol. 2016;21(11):2493–2502. 25904653 10.1177/1359105315580463

[49] Lenti MV , Cococcia S, Ghorayeb J, et al. Stigmatisation and resilience in inflammatory bowel disease. Intern Emerg Med. 2020;5(2):211–223. 10.1007/s11739-019-02268-0 PMC7054377 31893346

[50] Argent R , Daly A, Caulfield B. Patient involvement with home-based exercise programs: can connected health interventions influence adherence? JMIR Mhealth Uhealth. 2018;6(3):e47. 29496655 10.2196/mhealth.8518 PMC5856927

