# Supplementary material for: Exercise Perceptions and Experiences in Adults With Crohn’s Disease Following a Combined Impact and Resistance Training Program: A Qualitative Study
Source: Crohns Colitis 360. 2023 Mar 24;5(2):otad019. doi: 10.1093/crocol/otad019 (PMC10071982; doi:10.1093/crocol/otad019)
Supplement: otad019_suppl_Supplementary_Material [file otad019_suppl_supplementary_material.docx]

**Supplementary Material**

1. **Interview Topic Guide**

**Brief History**

- What were your presenting symptoms?
- When were you diagnosed? How long?
- How were you diagnosed?
- What is your history with Crohn’s disease- flare ups, problems related to your condition
- What medications have you tried?
- Any surgical procedures?
- Have you tried anything yourself to manage your Crohn’s disease?
- Has this method been successful? How?
- Do you experience abdominal pain or joint pain as a result of your condition? How long have you experienced this pain? Is there anything you do to reduce your pain? Is there anything you find makes it worse?

**Research Questions**

- What were your thoughts when you heard about the research opportunity? How did you hear about the research?
- From the information you received which of the following was most important and relevant when deciding to participate:

1. Invitation letter
2. Participant information sheet
3. Organisation (location/ timing of assessments)

- Was there any additional information you would have liked to receive that might have been helpful when you were deciding to participate?
- Why did you choose to participate in the research study?
- How did you find the study assessment at the hospital?
- What did you think about the setting at the hospital?
- How did you find the travel and length of the sessions?
- What was your relationship like with the research nurse and clinical investigators?
- How did you find the clinical assessments carried out at the hospital, such as the stool and blood samples and physical examination?
- How did you find the study assessments at the university?
- What did you think about the setting at the university?
- How did you find the travel and length of the sessions?
- What was your relationship like with the researcher and other investigators?
- How did you find the clinical assessments carried out at the university such as the bone mineral density scan, muscular performance tests and questionnaires

**Intervention Experience (EXERCISE GROUP ONLY)**

- What was your initial preference, control or exercise? Why was this you preference?
- What did you think when you were allocated to the exercise group?
- What did you expect from the exercise programme before you began?
- Could you tell me what you thought about the design of the exercise programme?
- How did you feel about completing three sessions a week? (*too often, just right, not enough*)
- How did you find the intensity of the programme?
- What did you think about the length of the session?
- What are your thoughts regarding the length of the programme?
- What are your thoughts on the type of exercise used?
- What are your thoughts about the setting of the exercise programme? Was it appropriate? Could it have been better? If so, how?
- Was there anything that made it hard for you’re to complete the exercise programme?
- What made you keep attending the sessions?
- Could you recommend are changes to the exercise programme if the study were to run again?
- In regards to the researcher delivering the exercise programme, can you tell me what your relationship was like? How did this relationship compare with your relationship with other healthcare professionals?
- Is there any aspect which could have been better? Do you think that the right sort of person delivered the intervention?

**Outcomes (EXERCISE GROUP ONLY)**

- Has the exercise intervention had any positive or negative changes on you, physical changes, anything you have noticed as a result of the exercise?
- Apart physical changes, how do you feel in yourself since completing the exercise programme?
- Do you see any changes in your condition after completing the exercise programme?
- Has it affected how you will manage you condition in the future?
- Has it changed your understanding of your condition?
- Do you see you condition differently than before?
- Abdominal pain, has the intervention affected the amount or severity of the abdominal pain you experienced before commencing the exercise programme?
- Joint pain, has the intervention affected the amount or severity of the abdominal pain you experienced before commencing the exercise programme?

**Acceptability** **(EXERCISE GROUP ONLY)**

- How acceptable do you think the exercise programme was?
- Would you recommend this sort of exercise programme to other people with Crohn’s disease? Why?
- Can you think of any reasons people with Crohn’s disease may not want to participate in this type of exercise? Or research study?
- Do you think this type of exercise training should be offered on the NHS for people with Crohn’s? If no, why not? If yes, what would the exercise training look like, what would you like to see?
- If exercise training wasn’t available on the NHS, would you be willing to pay for it and if so much would you be willing to pay?

1. **Individual Participant Characteristics**

| **Participant ID** | **Gender** | **Age** | **Employment Status** | **Age at Diagnosis** | **Duration of Diagnosis** *Years (Months)* | **CDAI Status** | **Group Allocation** |
| --- | --- | --- | --- | --- | --- | --- | --- |
| F001 | Male | 43 | Employed Full Time | 27 | 17 (216) | Inactive | Exercise |
| R002 | Female | 36 | Employed Full Time | 31 | 5 (66) | Inactive | Exercise |
| R005 | Female | 60 | Self-Employed | 30 | 30 (388) | Inactive | Control |
| R008 | Female | 71 | Retired | 31 | 40 (484) | Inactive | Control |
| R009 | Female | 48 | Employed Full Time | 17 | 31 (383) | Mildly Active | Exercise |
| R012 | Female | 47 | Self-Employed | 37 | 10 (142) | Mildly Active | Exercise |
| R013 | Female | 27 | Employed Full Time | 11 | 16 (192) | Inactive | Exercise |
| R014 | Female | 46 | Employed Full Time | 43 | 3 (36) | Inactive | Control |
| R015 | Female | 63 | Retired | 37 | 26 (312) | Inactive | Exercise |
| R016 | Female | 55 | Employed Full Time | 36 | 19 (228) | Inactive | Exercise |
| R017 | Female | 25 | Employed Full Time | 23 | 2 (24) | Inactive | Control |
| R018 | Male | 37 | Employed Full Time | 21 | 16 (192) | Inactive | Control |
| R021 | Female | 60 | Unemployed | 23 | 37 (444) | Mildly Active | Control |
| F002 | Female | 33 | Employed Part Time | 31 | 2 (18) | Inactive | Control |
| F007 | Female | 43 | Employed Part Time | 38 | 5 (60) | Mildly Active | Control |
| F008 | Male | 38 | Employed Full Time | 33 | 5 (60) | Inactive | Control |
| F009 | Female | 59 | Retired | 18 | 41 (492) | Mildly Active | Exercise |
| R024 | Male | 38 | Unemployed | 22 | 16 (192) | Mildly Active | Exercise |
| F015 | Male | 49 | Self-Employed | 32 | 17 (204) | Inactive | Exercise |
| R027 | Female | 44 | Self-Employed | 25 | 19 (228) | Inactive | Exercise |
| R028 | Female | 51 | Retired | 22 | 29 (348) | Inactive | Exercise |
| R030 | Male | 50 | Employed Full Time | 46 | 4 (48) | Inactive | Exercise |
| R031 | Female | 56 | Employed Part Time | 18 | 38 (456) | Mildly Active | Control |
| R032 | Male | 54 | Self-Employed | 33 | 21 (252) | Inactive | Exercise |
| R033 | Female | 60 | Retired | 50 | 10 (120) | Inactive | Control |
| R034 | Female | 36 | Employed Full Time | 31 | 5 (60) | Mildly Active | Control |
| R036 | Male | 59 | Self-Employed | 51 | 8 (96) | Inactive | Control |
| R037 | Female | 70 | Employed Part Time | 30 | 40 (484) | Inactive | Control |
| R038 | Female | 37 | Unemployed | 32 | 5 (60) | Inactive | Exercise |
| R039 | Female | 38 | Employed Full Time | 20 | 18 (216) | Mildly Active | Exercise |
| F018 | Female | 52 | Employed Part Time | 20 | 18 (216) | Inactive | Control |
| F019 | Female | 27 | Employed Full Time | 26 | 1 (12) | Inactive | Exercise |
| F020 | Male | 67 | Retired | 12 | 55 (660) | Mildly Active | Exercise |
| F021 | Male | 49 | Employed Full Time | 31 | 18 (216) | Inactive | Control |
| F022 | Male | 51 | Employed Full Time | 42 | 9 (108) | Inactive | Exercise |
| R041 | Female | 47 | Employed Full Time | 44 | 3 (36) | Inactive | Control |
| R044 | Male | 66 | Employed Full Time | 56 | 10 (120) | Mildly Active | Control |
| R045 | Female | 51 | Unemployed | 33 | 18 (216) | Inactive | Control |
| R046 | Female | 68 | Retired | 34 | 34 (408) | Inactive | Exercise |
| R048 | Male | 58 | Retired | 28 | 30 (388) | Mildly Active | Control |
| F023 | Female | 46 | Employed Full Time | 43 | 3 (36) | Mildly Active | Exercise |
| CDAI, Crohn’s Disease Activity Index | | | | | | |  |

1. **Additional Patient Quotes**

| **Theme** | **ID** | **Quote** |
| --- | --- | --- |
| Transition to inactivity | R002 | Just before and after I was diagnosed my body felt like it couldn’t do anything like it was just out of battery is the only way I can describe it [pause 3 seconds] erm and then being well I still couldn’t get back to it [pause 2 seconds] id be out of breath going up the stairs, doing housework and I’ve never been able to get back to what I used to do |
|  |  | I have a daughter now, fingers crossed it doesn’t happen to her but it might and I hope that any research that I would participate in now, 20 years down the line might make things better for her if she gets it and other people |
|  | R015 | I’d already been told on a number of occasions by the gym instructors there that I needed to be doing some resistance but when I tried to do it the machines hurt me and I didn’t know what I was doing wrong |
|  | R030 | I just thought you know, it would be nice to give something back, I thought I would help and I wanted to help because of everything that’s been done for me |
|  | R031 | I used to love going running and was in a running club but that all had to stop [pause 2 seconds]. Don’t get me wrong I went back to it but its hard because your not as good and it takes the enjoyment out of it slightly |
|  | R038 | It [exercise] just stops becoming your priority and I think that’s what happens with the condition, you get ill and that’s all your focus is on is getting better and if someone close gets ill everything around ya just gets dropped |
|  |  | I just hoped I could manage it because I haven’t done something properly since, well before I got the stoma so wanted to see what I could manage in safe place you know |
|  | R041 | I know I need the medicines but it would be good if other options were available, because you can get like fed up of taking them and even when they work like they might stop working or be a reason you need to stop it so yeah [pause 3 seconds] I think looking for alternative |
|  | R045 | Just to help out as much as possible [pause 2 seconds] if we don’t take part we will never know more about how we can help ourselves you know. We just wait to be told what might help instead of being involved in the process of helping ourselves |
|  | R048 | …I guess thinking about it now its [exercise] not something that you get asked about at appointments its always bowel related queries. Perhaps that needs to be looked at |
|  | F001 | I think I’ve realised that some form of exercise will benefit me |
|  | F009 | It would be good to discuss our results [of the main trial] with our consultants and so they can be made aware of the study and the results so they can offer different alternatives for people |
|  | F015 | I’ve played quite a lot of sport in the past and I had a bad flare, probably stressed with work and me fitness was erm lets say not great |
| Benefits of Exercise Participation | R002 | I think its actually given me quite a bit of erm confidence in a way like I actually feel like if I chose to go back to the gym like the spinning ive just signed up to, that I can actually keep up [laughs] but also not feel like people are looking at me thinking I can’t do it so yeah its been quite life changing in that aspect |
|  |  | …I had the pain for quite a few months but erm it just wasn’t really shifting. It would go away and come back, go away and come back and then I started the programme and it went and I was like wow |
|  |  | It has because its gotten me moving around more and given me a lot more erm strength [pause 4 seconds] which I think has help support my joints and I don’t think I would have got that if the programme wasn’t with the bands and was like erm oh, I dunno running on a treadmill |
|  | R012 | I think what I’ve noticed is that once I started the programme I then adapted my lifestyle as well, well I thought if I was doing this I needed to make sure I was eating better as well and I suppose I’ve drank more water, which is good for you, sleep, sleep as well I’m sleeping a lot better |
|  |  | I actually feel much better actually because I think it’s shown me I can do a certain amount of exercise and it’s not going to be detrimental it’s not going to going to make me excessively tired. You know if I am having a bad day sometimes I feel a lot better once I’ve done the exercise so you know in that respect I think it’s been good to show me I can do exercise and it’s not going to make me too tired, if anything its made me more energized and that’s not something I’d ever thought id say |
|  | R013 | I feel like I have more energy and when I went away on holiday but, the first time I went away I was really tired and I couldn’t do as much. But this time I went away we did quite a lot of walking and I was fine, still a little bit tired but not as bad so I feel like its helped with that and like I said before just general day to day activities like chores around the house. Eee I was so bad id leave whatever I couldn’t really do or didn’t have any energy to do and now they are all done in a day so I think that’s definitely help with it |
|  | R015 | I’m a stronger, sturdy more solid person than I ever used to be, I don’t know if I said that right what I mean is that I’m more solid on my feet. I just feel so great, I feel like the Michelin man |
|  |  | I just feel on top of the world to be truthful with you, I just feel as fit as a fiddle |
|  |  | Mentally strong, happy, really good, I’m in a really good place, really good place just feel over the moon with what I’ve achieved I, I really am [pause 3 seconds] can we do it again [laughs] |
|  |  | I’ve had the condition for so long I don’t think I’ll ever see it differently. Em, I just think because I’ve coped it with and its my coping mechanism to see the condition the way I do em, but it’s really good to see that I can do things to help myself |
|  | R024 | I’ve got more muscle definition which I haven’t had since I was younger. So yes definitely positive changes, the biceps I’m definitely grateful for [laughs] |
|  |  | The severity of the pain I had before I would say has definitely eased so being able to do some exercise to help it is definitely the way forward |
|  |  | I thought I was indesctrubale until the condition and all of a sudden your like, well your stripped of what you can do really. So being able to do something like this when I’ve gone through those peaks of not feeling well is something I’m going to continue |
|  |  | It’s a big difference once I found out I had the condition it was good that I had a diagnosis but that was it it was take these meds and see if they work and I just like felt helpless as to what I could do to help apart from avoid things that make it worse you know |
|  |  | It’s taught me what I need to do to achieve what I have and it has also helped me get back to a stage in my life which I haven’t been for a long time |
|  |  | …I feel like I’m so energised and full of life again its great |
|  | R027 | No changes in my condition I don’t think its done anything bowel wise…it’s not made me like go to the toilet anymore, or feel like I need to rush to the loo and I’ve not noticed any pain but I’ve never had loads of pain its normally just when I flare up I get it |
|  |  | It went so quickly I couldn’t believe when you said it was at the end [laughs] but definitely feel erm what’s the word, like perkier and bouncy, that’s probably not what I wanted to say but it’s the best way to describe it |
|  | R028 | Better mood, feel good and positive in my mental health |
|  | R030 | It’s been quite life changing |
|  |  | It’s been quite an eye opener with how many people get the bone density and that you can do something that’s, well free, or well not too expensive, I dunno how much it costs for the er bands but its not an expensive thing and we aren’t being told this or told how we can do stuff to er make it go away or better |
|  | R032 | You know I can actually walk my dog now without getting tired, he doesn’t know what’s going on getting longer walks [laughs] |
|  |  | I feel positive, I feel overwhelmed to be honest with you [RESEARCHERS NAME] really with how good I feel, never thought I’d ever feel like this again after being diagnosed |
|  |  | You get a better understanding of your condition, you learn things that a say book doesn’t tell you. You do that thing when your diagnosed where you read everything online about Crohn’s and its all the bad stuff, never a positive outcome |
|  |  | Yes, because okay you know you have a flare up you have a bad belly you get diarrohea and all the other joyful side effects, but it’s the other bits and pieces you don’t know are going on behind the scenes like aching joints, bad bones which is affected by your Crohn’s. Then the meds, the steroids although they help don’t help the erm the other bits so now I know and have a better understanding of how I can help myself |
|  | R038 | I’m sleeping a lot better but not napping during the day [pause 2 seconds] at first I thought it was going to make me tired and well worse but I’ve been really surprised at how its improved my tiredness [pause 2 seconds] which then gives me the time to do more stuff. I mean I stopped working a few years back because I felt as though it sort of won and having the ups and downs with it and the stress from work not understanding, it was better to just not do it [pause 2 seconds] but now I feel like I could do more and potentially look at er well going back to work or doing something during the day instead of just napping |
|  |  | I feel better mentally |
|  |  | I know what I’m capable of and I can do it and that I just need to keep that up and it will benefit me so I guess I’ve got more awareness and more knowledge that I know it can work. |
|  | R039 | I think it’s given well you’ve taught me what I can do that doesn’t make my stomach bad, but also what I need to do to make sure I get all the er benefits physical and mental changes so I think it will be something I carry on to see those changes more and hopefully stop any other problems starting |
|  |  | I never knew the level or well how much the condition can cause problems it’s been a new learning curve for me |
|  | R046 | It’s been one of the best things I did and I’ve learnt so much about myself and yes its just been a very erm good and insightful for me and I feel hugely proud of myself for doing and giving everything in to the programme |
|  |  | I can do things to help myself that doesn’t erm cause my condition any problems but actually makes me feel better about myself and can prevent the bone loss |
|  | F009 | I think the results that I can see in myself and how I feel in myself has been very positive, I feel fitter, taller in myself and stronger |
|  |  | I think initially it was whether I could physically do it, was it going to going to make my bowel worse after it taking so long to get under it erm under well control really and the fatigue was a huge factor for me not sure whether it would make me worse and tire me out |
|  |  | It’s the best I’ve felt in years |
|  |  | Because I’ve had the condition for so long there’s nothing that can shock me anymore with the disease. There’s always new information that comes out or a condition that’s been associated with having Crohn’s |
|  |  | I think that I've done it has given me hope and confidence in my ability |
|  | F015 | My nurse said this as well that I never felt or looked as good in me life…me blood pressure as well that’s come down and I feel a lot better in meself |
|  |  | Even me nurse says I look better and I’ve had so many compliments from people about how great I look |
|  | F019 | I feel great in that [pause 2 seconds] think it always makes you feel better when you look better and I’ve noticed more in the last few months that ive started like er hunching over cos of me pain but I’ve noticed I’m much straighter |
|  |  | Think im still getting used to having it and like er recognising what’s good and what’s bad like me twinges and that [pause 3 seconds] the bloating used to make me feel so er, well its not a good look so having that helped is huge to me and when you feel like you look good your mindset is, well it follows |
|  | F020 | I have more willingness to get up |
|  |  | Because I have had the condition so long I feel as though I already understand it a lot better than someone who was maybe perhaps more recently diagnosed or even more well with it [pause 3 seconds] I’ve been, well I wouldn’t say poorly but being on 12 hour feeds a day because of what the condition has done to me does open your eyes to it more |
|  |  | With the length of time I’ve had Crohn’s, I’ve been diagnosed since I was 12 so I think its been along progress of understanding my condition and coming to terms with what I can do and can’t do, physically and mentally. I wouldn’t say the programme has made me see it differently before |
|  | F022 | Feel better in myself just generally more energy |
|  |  | I was obviously very motivated to do it because I knew what I was getting from it was positive |
|  |  | If anything it’s made me realise what exercises I need to do to achieve things |
|  | F023 | I see it more positively I think knowing that although it may get the better of me sometimes I can do stuff and it wont stop me, so yeah I think it has made me realise that I’m someone that has Crohn’s but that’s not the end of the world and you can still enjoy yourself even if times are hard sometimes |
|  |  | Now I know the bands and how to use them and know it will help my bones I think it has provided me with that knowledge to avoid getting any bone issues. But you know even if it doesn’t help my bones I’ve enjoyed doing them it’s been fun so I’d do it regardless |
| Barriers and Facilitators to Participation | F001 | There were other things that sometimes took priority, especially my daughter with health issues |
|  |  | I would have preferred more time coming to the University, and having more one to one sessions as it was actually really helpful for me to have that motivation and talk to someone and just yeah I think that was better than doing them at home |
|  | F009 | I was getting a lot from the exercises, I wanted to come in and see my progress, erm I think I was just getting a lot from it |
|  | F015 | I would have been happy to go on for another 26 weeks [laughs] and carry it on and feel the way I do |
|  | F019 | I thought like this kind of thing is going to help someone else further down the line if they’ve diagnosed with Crohn’s, or helping exercise to get the energy up. I just had that in the back of me mind that I’m going to help someone else and I was feeling better in meself |
|  | F020 | I wanted to keep improving [laughs] I was, well am getting so much out of it and my nurses have noticed that and the muscle mass I now have so I wanted to continue with graining the benefits |
|  | F022 | I’ve got kids and erm time, time’s hard and you’re juggling stuff so just being able to do something at home with the bands was obviously appealing, very appealing to me |
|  |  | I thought it would be in a gym or something like that. |
|  | F023 | I think if if I was totally doing it at home, it probably would have like maybe I wouldn't have done it. |
|  |  | It's been good seeing our progress, really, because I’ve achieved all my goals I set myself you know |
|  |  | You've always told us what's going to happen and things like that, it just felt very at ease and very calming, I know that sounds really weird but I just felt at ease throughout the entire process |
|  |  | I was seeing the benefits and getting a confidence boost from all the compliments from my friends. And it's like it's hopefully helping other people to make them better in the future |
|  |  | I think for some reason I maybe saw it as a gym class and doing it like that |
|  | R002 | It felt different to like a gym it was abit more personable and just better in a way because you were interested to talk about the condition and I think that helped. So from someone who had the condition, its really nice to share your experiences and talk about it as its not something that you come across everyday, I don’t know anyone else with the condition so yeah I found it nice to talk to someone and sort of share my frustrations [laughs] and you understand those things |
|  | R009 | Yeah [pause 3 seconds] at home I think you start to loose the routine after a while and end up getting home after a long day and just put the TV on, then feel a little guilty knowing you should be doing something |
|  |  | Did it make a difference to us? Yes, it did erm, but what I think is, what I hoped to do is more than what I could physically manage with everything else just general everyday activities and I can do that, I’ve achieved that. |
|  |  | I didn’t have to explain to you anything about a stoma or surgery or Crohn’s or anything like that you just understood and that was really nice to have. I think with the condition you always feel like you have to explain yourself or try to get people to understand |
|  |  | I think for me I would have liked to do more sessions with you because I felt more motivated and I enjoyed our chats and having it as part of my routine |
|  | R012 | It was that thing of not wanting, not wanting to let you down and being responsible with contributing to the trial |
|  | R013 | To see the outcome really, I wanted to see whether I would like get stronger and feel better about myself after my operation and just see what I was able to do as well. |
|  |  | I work long shifts and when I got home I just was conked out |
|  | R016 | I was getting benefits out of it, I felt better at work and I enjoyed it was nice to come in after work and look forward to something |
|  | R024 | It was also helpful to attend because you provided that reassurance that everything was being doing correct and also whether I needed to change bands or go up sets and reps so I think it was helpful to have that. And I suppose as well from a social aspect its nice to do the exercises alongside someone or just even talk to someone while doing it about Crohns’ as well |
|  |  | Had the off day where I lost a bit of motivation however I felt that came back after the sessions at the university because you just felt like a new lease in a way. |
|  |  | I was really seeing what my potential was and wanted to do more so 6 months was just right I think for what the goals of the research are |
|  | R027 | I was getting a lot out of it I could see my body changing and I was getting fitter so win win [laughs] |
|  |  | I’ve always believed being fit like that helps but then it’s a hard thing to do when you are poorly. Which is why I think it’s important to do it at home because you can continue it even if you’re not feeling 100% you can do it even if your flaring because you have the toilet just there |
|  |  | It was good to see where everything is done |
|  | R030 | I didn’t know what to expect I didn’t think it would be as intense as what it was and involve as much of that er much but then when I think more about it I don’t know why I didn’t expect that. It wasn’t gonna be a walk in the park really was it [laughs] but everything was done for a reason and I know why and well I can see why |
|  | R032 | I was very adamant to give my all towards this research as I think it’s just so important |
|  |  | I didn’t have any problems with it what so ever was refreshing to do something that I’ve never tried before and be pleasantly surprised at how much I actually prefer the bands over using weights |
|  | R034 | I know I was in the group that didn’t exercise and stuff but it was just to see if I can do it and if I could would it help my Crohn’s |
|  | R037 | being able to give something back, and help other people with the condition. I think when I was diagnosed there wasn’t much research or much known about the condition, so yeah the more we can do the more we help the next generations |
|  | R038 | I actually enjoyed the stuff we did and it was interesting learning about the tests and examines and stuff |
|  |  | It was good to talk to someone with the condition who knew about what I was going through, as I think its easier for them to understand like how your actually feeling and is just willing to listen as well it was enjoyable to talk to someone and not be judged |
|  | R039 | You can do it in your own home so you can adapt to fit it in and its not like you need any heavy equipment and you can do the amount of repetitions to what you can do so its very easily done. |
|  |  | I could see myself getting stronger and just feeling better in myself that I wanted to continue feeling that way |
|  |  | No judgements, no one, well there’s nothing that you feel like if you don’t sort of do that you’re going to let people down |
|  |  | The university was great yeah, kind of what I expected with the classrooms and stuff, it was nice to see the er the labs on where the tests are done and they look a lot friendlier than the ones at the hospital |
|  | R041 | I think there should be more studies into other alternatives to medicines and things |
|  |  | I was open straight away as soon as I was like seen it I was asking my doctor about it and then I think I spoke to one of the research nurses and yeah [pause 4 seconds] just really excited for something new |
|  | R045 | I was really pleased to see more research being done, I think for me I’m very open to looking to alternative options |
|  | R046 | I’m going to continue doing, the exercises are so easy to do and very easy to do in my home as well so it will be something that I continue to do in my day to day life yes |
| Perceptions of programme design | R002 | I also liked that the toilets were just outside the room as I always feel when im exercising I find myself sometimes needing to use the toilet quite urgently so it was good that that was there |
|  |  | The book that you gave us was really helpful, although we had the sessions as well it was really helpful and informative and being able to do them at home after having those sessions was great |
|  |  | It was good that we started off with a low coloured band, I think it was yellow and erm yeah it could be made harder or easier depending on you sort of ability so yeah I really think it was well thought through |
|  |  | I think using resistance bands is a safer option than to start lifting weights. |
|  |  | It was gradual it wasn’t just full on it was a gradual build up which was good as id never done erm the strength training before so was abit nervous at first but after the first session, although I ached, I felt great like I’d never thought id be on the blue band after 6 months it was my sort of goal [laughs] to get like to the next colour or set |
|  | R009 | I think it should be a part of your treatment pathway if you want to say erm for anybody. And you know if you achieve what you would set out to find out is once you’re diagnosed you’ve got, you could have a poor bone density but you could do X, Y and Z it needs to be well it needs to change your diet, have this immunosuppressant and look at this kind of exercise you know |
|  | R012 | So for me I found it absolutely fine for me it was also at my level if that makes sense but always challenging, don’t think there was ever a session I found really easy. |
|  | R013 | Well at first especially the day after when I was abit sore I thought wow that was hard but as you went on and your body got used to it it was good as you did it at your level so yeah at first I was taken back about how much my body was aching then thinking I’m never going to go up these colour bands. But then it got easier, well not in an easy way but you ached less and you got comfortable with er the er techniques |
|  |  | I take stool samples in for appointments and have blood tests all the time so nothing new |
|  | R015 | I’ve tried the machines and I just find I’m just knocked for 2 or 3 days and that’s just no good for me so I’m just keeping on with the bands |
|  | R016 | I’d never done it before it was brand new to me but I think I prefer doing the exercises we did because it was at my pace and I could alter how many reps and how hard the bands were |
|  |  | It absolutely flew by I cannot believe it’s been that long and like I would have done it for another 6 months |
|  | R024 | Maybe if there was a group of people doing the sessions that way you could also talk to other people with the condition and share sort of tips with how they were doing the er the exercises |
|  |  | I just think if you gave someone this after a flare it would make them feel better and they may even go longer without flaring up you know. I think exactly what we did would be what I want to see |
|  |  | I have continued doing the exercises since the programme finished. Its taught me what I need to do to achieve what I have and it has also helped me get back to a stage in my life which I haven’t been for a long time |
|  | R027 | The bands and do it at your own level really. I quite liked that as you set yourself the goal of doing so many reps or getting to the next band colour so it was a motivator for me to have |
|  |  | It was fine, normal exercise class length really don’t think you could make it any shorter because then you just wouldn’t have the same er well my muscles wouldn’t have grown [laughs] but if it was any longer I don’t think that would do much more or be helpful really as I was tired as well [laughs]” |
|  | R028 | I’ll do more exercise because I know its fine for my bowels and has had positive effects, learned a lot |
|  | R030 | I think it would be perfect for a lot of people. Well I think even GPs being encouraged to ask, just in general to anyone who is overweight or not feeling good about themselves this will make your mindset better and look better definitely |
|  |  | I was very surprised to find them better than using weights like they were just so easy and simple and you know I enjoyed doing them thoroughly |
|  | R032 | It was just right you had the beginning where you, well I was shocked at how my muscles were the next day don’t think they had been used for years. Then towards the end you had passed all expectations of where you thought you’d get to. |
|  | R038 | I think I would like to see it on the NHS yes it could be really helpful to people. I think it would be quite good range of exercises then they could be adapted person to person you know if you had a bigger booklet say, then somebody that picked the best five out of the booklet for that person, then maybe you could alternate it when they next came in or later on. So I guess yeah id like to see a good range |
|  | R039 | The resistance bands were really good, as they were really strenuous to the point where you couldn’t do stuff, well at first it was difficult but after a few sessions it seemed to get easier |
|  | R044 | With the samples, nothing you don’t already have to do [laughs], I erm get my bloods done every time I see my doctor |
|  | R046 | I think that would be so helpful, I think it would save the NHS a lot of money as well with all the other problems this disease causes |
|  |  | I didn’t know how intense it could be but it was good because you could go at your own pace which I thinks really important. At first I thought the intensity was too hard for me to do but again after the first weeks you just get used to it and then end up doing more and more and the stuff you did at the beginning is nothing on what you are able to do now |
|  |  | I did like the book you gave us at the beginning because we went through the exercises in detail and how to make them harder |
|  | F001 | I feel like it was something that helped me bounce back quicker. I know there’s no running back from surgery I get that but I do feel like this helped me get to a stage in my life I didn’t think id get back to for a lot longer |
|  | F002 | I liked is that they were spread out and you weren’t having to come back often and everything was explained so I knew what was going on like there wasn’t any point during the study were I felt I didn’t know what was going on |
|  | F007 | Easy to understand and everything was explained well erm so yeah I knew what I was going to be doing and why which was really helpful like [pause 3 seconds] you sometimes go to the hospital and you get bloods and stuff done and, [pause 3 seconds], and never really know why your getting them done so yeah was just interesting to know why I was getting the scan and that was for my bones |
|  | F009 | I really liked the fact you came and met me and pointed out the toilets straight away it kind of made me feel at ease |
|  |  | It’s all at your own pace and ability, you have different stages in the programme |
|  | F019 | The exercises were very user friendly, very and could be done anywhere at any time. It was actually very well thought through |
|  | F015 | It was also good that you made it easier when I couldn’t manage, long hours at work, so I still did it and felt good after because I was able to do it |
|  |  | I would have continued to do it for ever really, I’ve continued to do it after the erm the erm programme stopped |
|  |  | The book and everything was straightforward. It explained everything so in terms of that it was perfect, you know what I mean it was a good, a good mix up of different er, using different parts of the body. |
|  |  | Just really enjoyed the muscle tests it was like a challenge to myself to get better |
|  |  | I struggled to do it at home, think with work and stuff I would have liked more sessions as I think sometimes it was hard to motivate yourself, well I thought it was great doing the sessions in the University so I would have liked more |
|  | F022 | I much preferred it, coming in and having the sessions with you because it was more motivating if it makes any sense. I’m there to do this, I’m there for a reason boom, boom … I’ll do it |
|  |  | You always did the sessions around me and my availability even did the sessions on weekends and late nights to accommodate for picking up the kids and working. So yes it was very well organised |
|  |  | The bone scanner was fantastic to have that was great it was something I heard about with Crohn’s but never knew the extent |
|  | F023 | It's been good seeing our progress, really, because I’ve achieved all my goals I set myself you know |
|  |  | Surprising to be honest, I never thought id be able to use the bands because I’m quite weak in my upper body but I was pleasantly surprised with how much I could manage and how adaptable the equipment and exercises were around my ability. |
|  |  | I really enjoyed the exercises and I’m going to keep them up. Now I’ve got the bands and know what I need to do I’m definitely going to keep it up…” |
